# Biomechanical effects of varus stem alignment on a cementless metadiaphyseal anchoring hip stem: a biomechanical investigation

**DOI:** 10.1186/s42836-026-00413-7

**Published:** 2026-07-03

**Authors:** Philipp Kastner, Matthias Luger, Moritz Kraus, Ivan Zderic, Boyko Gueorguiev, Tobias Gotterbarm, Clemens Schopper

**Affiliations:** 1 Department for Orthopaedics and Traumatology, Kepler University Hospital GmbH, Johannes Kepler University Linz, Krankenhausstrasse 9, Altenberger Strasse 69, 4040 Linz, Austria; 2 AO Research Institute Davos, Clavadelerstrasse 8, 7270 Davos, Switzerland; 3 Department for Trauma Surgery, University Hospital Zurich, University Zurich, Rämistrasse 100, 8091 Zurich, Switzerland

**Keywords:** Varus malposition, Cementless hip stem, Axial stability, Biomechanical cadaveric testing

## Abstract

**Background:**

Minimally invasive surgical approaches have become standard in cementless hip arthroplasty; however, the recent literature still lacks biomechanical evidence regarding the effects of stem malpositioning. This study aimed to biomechanically evaluate the stability of a cementless metadiaphyseal anchoring stem implanted with a varus malposition versus a neutral alignment.

**Methods:**

Twenty paired human cadaveric femora were assigned pairwise for stem implantation featuring either 8° varus malposition (Group 1) or neutral orientation (Group 2). All specimens underwent quasi-static testing and progressively increasing cyclic loading to failure with monitoring via motion tracking.

**Results:**

Axial stiffness in Group 1 was significantly lower than in Group 2, *p* = 0.023. Implant size in Group 1 was significantly smaller than that in Group 2, *p* = 0.002. Load at 0.15 mm stem subsidence and cycles to 0.15 mm subsidence in Group 1 were not significantly different compared to Group 2, *p* = 0.214. Load at 1 mm subsidence and cycles to 1 mm subsidence in Group 1 were significantly higher than those in Group 2, *p* = 0.022.

**Conclusion:**

An 8° varus-aligned cementless metadiaphyseal anchoring hip stem demonstrates superior load-bearing capacity with higher loads and numbers of cycles until reaching defined subsidence thresholds under dynamic loading, as compared to neutral alignment. These results demonstrate the biomechanical tolerance of unintended intraoperative varus malalignment, but do not support or recommend intentional varus stem positioning.

## Introduction

Minimally invasive surgical approaches have become a standard procedure in orthopedic surgery [1–3]. Especially in the field of total hip arthroplasty, the advantage of soft tissue sparing procedures is evident. Reduced intraoperative blood loss and time effort, shorter hospital stays, faster recovery within the first 3 postoperative months, better function, and reduced pain are the pearls of minimally invasive surgical approaches for total hip arthroplasty [2, 4]. Nevertheless, these advantages become evident in the first 3 postoperative months only, as their outcome assimilates with the outcome of classic open approaches, leading to a similar result from that time point on [5].

The pitfalls of the minimally invasive procedure in total hip arthroplasty can mainly be related to reduced intraoperative view over the surgical site [6]. This issue can lead to malposition of both cup and stem components [6–8]. Whereas malposition of the cup leads mainly to stability issues as a consequence of offset irregularities, aberrant cup inclination, and cup version, malposition of the stem primarily leads to dimension problems in terms of undersizing and, consequently, suspected vertical instability [9]. The reduced intraoperative view during stem implantation in e. g., an obese body configuration of the patient can result in a lateralized cup position, a conservatively resected femoral neck, and an insufficiently performed surgical release [10–12]. These factors hinder adequate proximal femoral elevation, increasing the risk of varus malposition during femoral broaching regardless of stem design or anchoring philosophy [13]. The main issue for the surgeon consists of the steep angle that must be passed through with the broacher. As a side effect of the degree of varus malposition, the risk of implant undersizing and intraoperative fractures arises too [14, 15]. Implant undersizing with regard to the stem component leads to an aberrant cortical fill index and is suspected to result in vertical instability leading to sintering and secondary joint instability [13, 14].

Recent literature still lacks biomechanical evidence about the behavior of a cementless metadiaphyseal stem implanted in a defined varus malposition versus a neutral alignment. Referring to defined values for micro- and macro-instability, the cut-offs for vertical instability of cementless hip stems are well known [16]. Based on the increasing use of cementless metadiaphyseal anchoring hip stems, this biomechanical study was set up to investigate the influence of a defined varus malposition of the stem—related to a consecutive undersizing—in comparison to a neutrally aligned implant.

## Materials and methods

### Specimens and study groups

The specimens used in this study were acquired from Science Care (Phoenix, AZ, USA). All donors provided their written informed consent inherent within the donation of the anatomical gift statement during their lifetime. The study was approved by the internal review board. Ten pairs of fresh-frozen (− 20 °C) human cadaveric femora with intact soft tissues from 3 female and 7 male donors with a mean age of 69.6 years (range 42–84 years) and an average bodyweight of 79.8 kg (range 44–109 kg) were used. No bony lesions, malignancy, or metabolic diseases were identified. A pairwise anatomical assignment to two study groups—Neutral Group and Varus Group—consisting of 10 femora each (*n* = 10) and featuring a homogeneous distribution of left and right anatomical sites was performed. A priori power analysis resulted in a minimum sample size of 9 specimens per group for a statistical power of 0.8 at a level of significance 0.05 under the assumption of normality of data distribution and a standard deviation in each group not bigger than 90% of the difference in mean values between the groups. Whereas in the Neutral Group the stem was implanted according to the manufacturer’s guidelines in a neutral position regarding its varus/valgus orientation, in the Varus Group the implantation was performed with an inherent 8° varus malposition.

### Surgical technique and specimens preparation

**Fig. 1:**
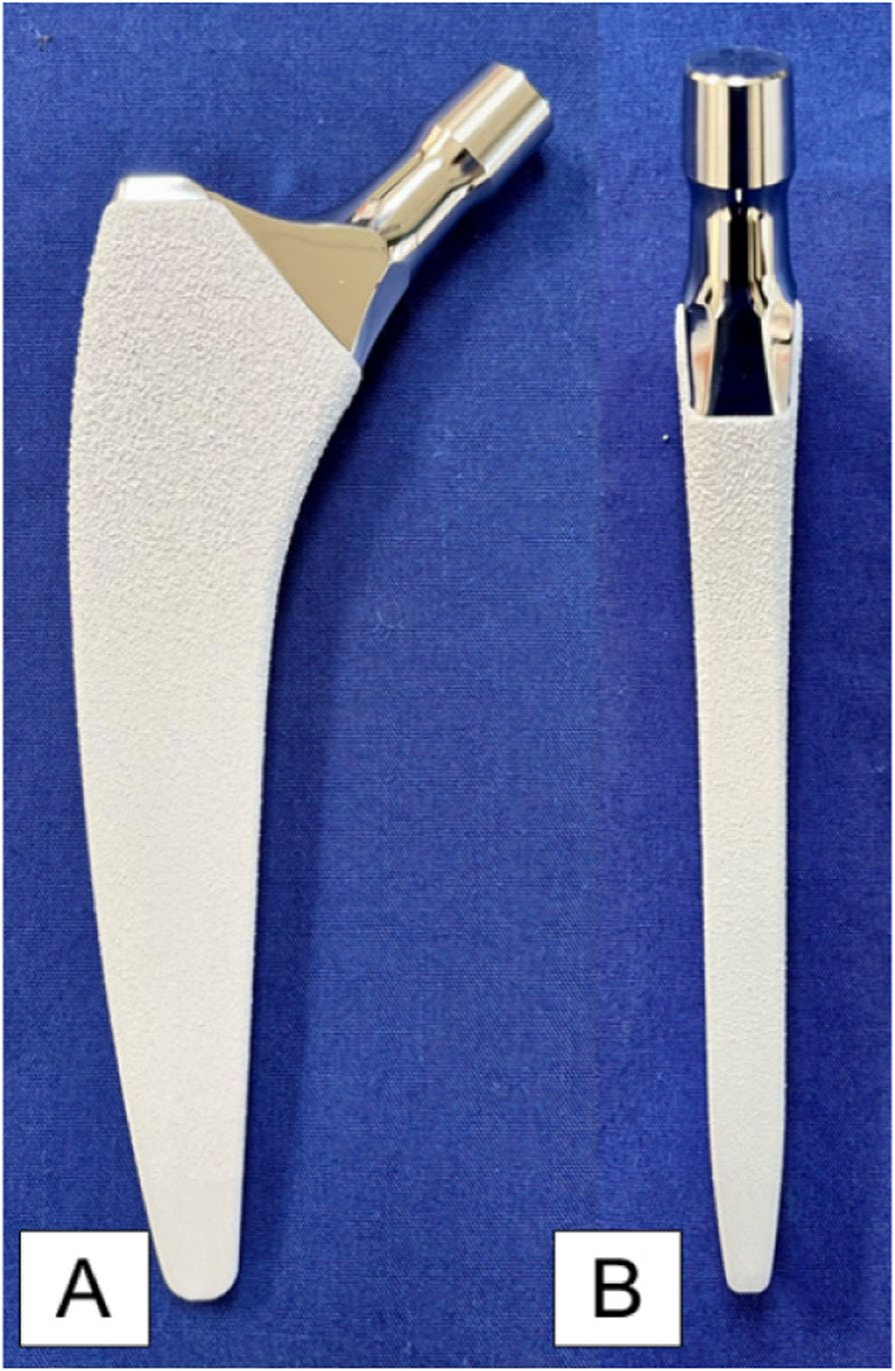
Anteroposterior (**A**) and side (**B**) views of a calcium phosphate titanium plasma-coated diaphyseal anchoring press-fit stem (ANA.NOVA alpha stem, Ti6Al4V alloy, 135° CCCD, double conus design, polished taper; ImplanTec, Mödling, Austria)

All specimens were thawed at room temperature for 24 h, and soft tissue was stripped off by an experienced surgeon. Anteroposterior X-rays were taken prior to implantation, and a planning program (mediCAD, Altdorf, Germany) was used to determine the respective anticipated size of the implant for each separate specimen. A calcium phosphate titanium plasma-coated diaphyseal anchoring press-fit stem (ANA.NOVA alpha stem, Ti6Al4V alloy, 135° centrum-caput-collum-diaphyseal (CCCD) angle, double conus design, polished taper; ImplanTec, Mödling, Austria) was chosen for implantation (Fig. 1).

**Fig. 2:**
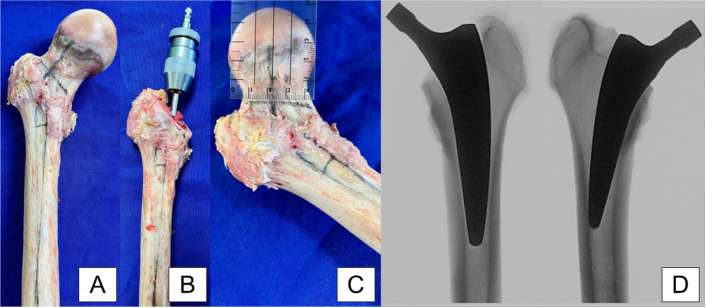
Specimen preparation. **A** The anatomical femoral shaft axis was defined arithmetically, and an 8° varus deviation was marked. **B** The implant bed was predrilled along the defined direction to guarantee an implant position as close as possible to the aimed orientation. **C** The femoral neck axis was defined arithmetically; the resection was performed in 90° orientation to the femoral neck axis, starting from the turning point of the greater trochanter and the femoral neck. **D** Anteroposterior X-rays of two exemplified implanted specimens from the Neutral Group (left) and Varus Group (right)

A new implant with an intact calcium phosphate coating was available for every separate specimen. The stems were implanted under X-ray control using a press-fit technique according to the manufacturer’s technical guidelines. A femoral neck osteotomy was set at an angle of 90° to the femoral neck axis, starting from the turning point of the greater trochanter and femoral neck. The implantation depth of the stem was chosen as close as possible to the level of the piriformis fossa side, the latter being symmetrical for the paired specimens. The anteversion of the implant was set at the physiologic value of each specimen by orientation to the calcar fulcrum. For the selection of different implant angles in the 2 groups, the anatomical axis of the femoral shaft was defined by the calculation of its diameter. In the Neutral Group, the stem was implanted in line with the anatomical axis. In Varus Group, the stem was implanted with 8° varus deviation with respect to the anatomical axis. To guarantee implant position as close as possible to the aimed orientation, in-line predrilling was performed in each specimen (Fig. 2). All femora were resected to the same length under fluoroscopic control and embedded in a 65 mm deep cylindrical Polymethylmethacrylate (PMMA, SCS-Beracryl D28, Suter Kunststoffe AG, Fraubrunnen, Switzerland) base. Markers were set to the femoral shaft and the stem for monitoring of their relative movements via motion tracking.

### Biomechanical testing

**Fig. 3:**
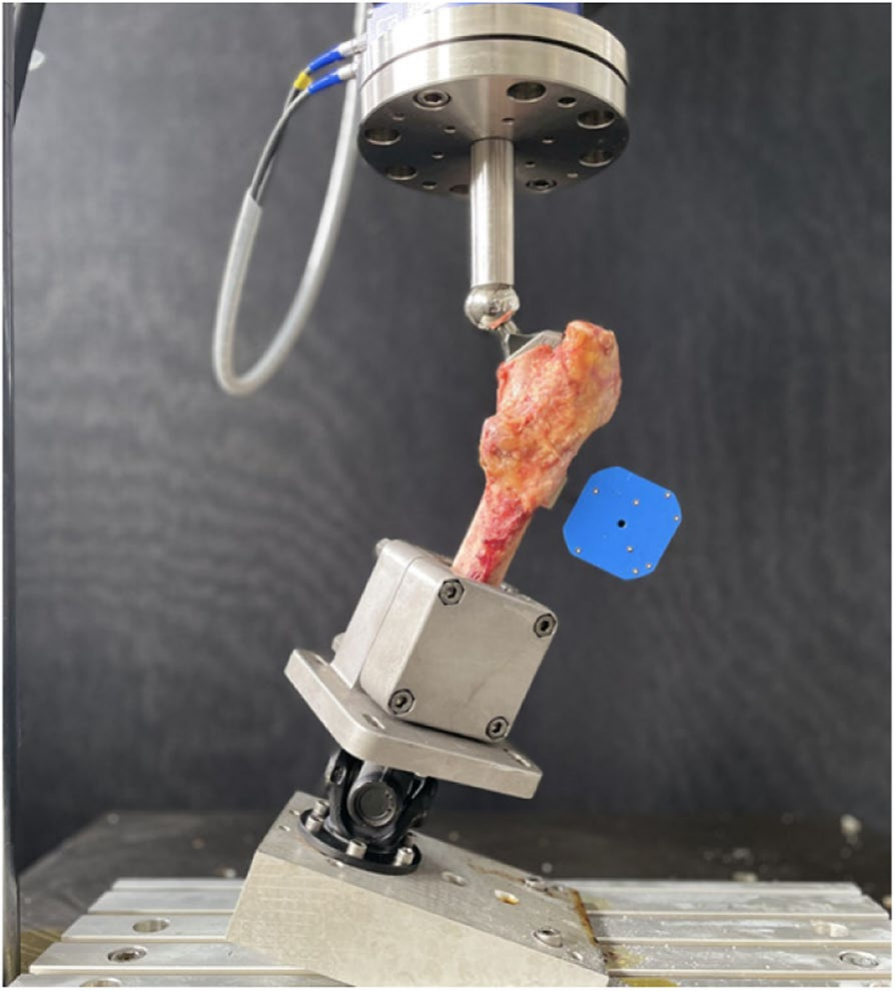
Setup with a specimen equipped with markers for motion tracking and mounted for biomechanical testing

Biomechanical testing was performed on a servo-hydraulic test system, Bionix 858 (MTS Systems, Eden Prairie, MN, USA) with a 25 kN/250 Nm load cell. The test setup and loading protocol were adopted from previous studies. All specimens were mounted in 20° adduction to the axis of the machine actuator. Proximal load transfer was performed via a 36 mm steel head with an indenter. A cardan joint was installed distally (Fig. 3).

The loading protocol considered an initial quasi-static and a subsequent cyclic loading phase. The quasi-static phase consisted of a ramped compression loading from 50 to 200 N, performed 3 times at a rate of 15 N/s. The cyclic phase implemented a double-peaked physiological compression profile of each cycle and a test rate of 2 Hz. Whereas the valley load of the cycles was kept at a constant level of 200 N during testing, their peak load, starting at 500 N (cycle 1), increased gradually by 0.1 N/cycle until the catastrophic specimen’s failure, the latter being secured by a test stop criterion of 30 mm displacement of the machine actuator. The method of dynamic biomechanical testing via progressively increasing cyclic loading has been proved as useful in previous studies [17].

### Data acquisition and analysis

Machine data in terms of axial displacement and axial load were acquired at a rate of 128 Hz by the machine controllers. Axial stiffness was calculated from the linear slope of the third load-displacement ramp during quasi-static testing, taking account of the specimen’s settling. Based on both machine and motion tracking data during cyclic loading, the peak loads related to 0.15 mm and 1 mm subsidence of the stem relative to the femoral shaft—both set as thresholds indicating initiation of corresponding micro- and visible macro loosening [16]—were evaluated and defined as load at 0.15 mm subsidence and load at 1 mm subsidence, respectively. In addition, the corresponding numbers of cycles until reaching these subsidences were calculated and defined as cycles to 0.15 mm subsidence and cycles to 1 mm subsidence. Finally, the peak load related to the rapid change of the load–displacement curve during cyclic loading—indicating definitive loss of stability—was evaluated and defined as load at catastrophic failure, along with the corresponding numbers of cycles—calculated and defined as cycles to catastrophic failure.

Statistical analysis among the parameters of interest was performed using SPSS software (V29, IBM SPSS Statistics, Armonk, NY, USA). Normality of data distribution and homogeneity of variances were checked and confirmed with Shapiro–Wilk and Levene’s tests, respectively. Paired-Samples T-test was used to screen and detect significant differences between the groups. The level of significance was set at 0.05 for all statistical tests.

## Results

Results for axial stiffness, stem inclination, implant size, along with loads and numbers of cycles related to the predefined stem subsidences and catastrophic failure are summarized in Table 1. Axial stiffness in the Neutral Group was significantly higher than in the Varus Group, *p* = 0.023. The Varus angle in Neutral Group (target value 0.0°) was significantly smaller than that in Varus Group (target value 8.0°), *p* < 0.001.

**Table 1:** Results for axial stiffness, stem inclination, and implant size, along with loads and numbers of cycles related to the predefined stem subsidences and catastrophic failure, are presented in terms of mean value and standard deviation for each group, together with the *p*-values from the statistical comparisons between the groups

Parameter	Group		*p*-value
	**Neutral**	**Varus**	
Axial stiffness (N/mm)	2,160 (340)	1,770 (840)	0.023
Varus angle (°)	0.7 (2.4)	8.1 (1.5)	< 0.001
Implant size	4.9 (1.5)	3.2 (1.2)	0.002
Load at 0.15 mm subsidence (N)	1,240 (551)	1,939 (1488)	0.214
Cycles to 0.15 mm subsidence	7,396 (5507)	14,392 (14,879)	0.214
Load at 1 mm subsidence (N)	2,248 (921)	2,858 (825)	0.022
Cycles to 1 mm subsidence	17,477 (9297)	23,518 (8249)	0.022
Load at catastrophic failure (N)	3,845 (1,249)	4,362 (1,518)	0.018
Cycles to catastrophic failure	33,454 (12,494)	38,615 (15,184)	0.018

**Fig. 4:**
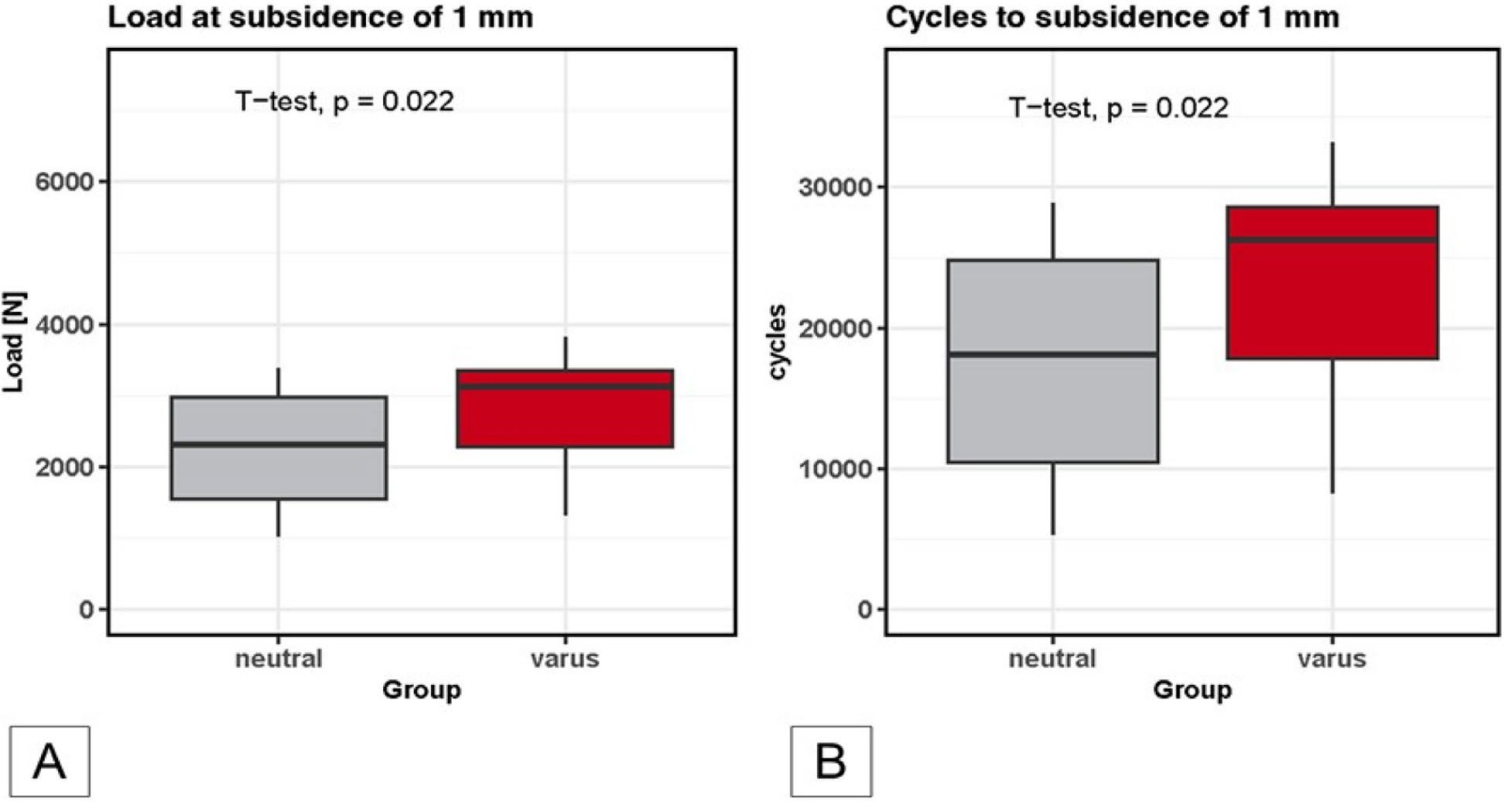
Boxplots visualising load at 1 mm subsidence (**A**) and cycles to 1 mm subsidence (**B**) in each separate group

**Fig. 5:**
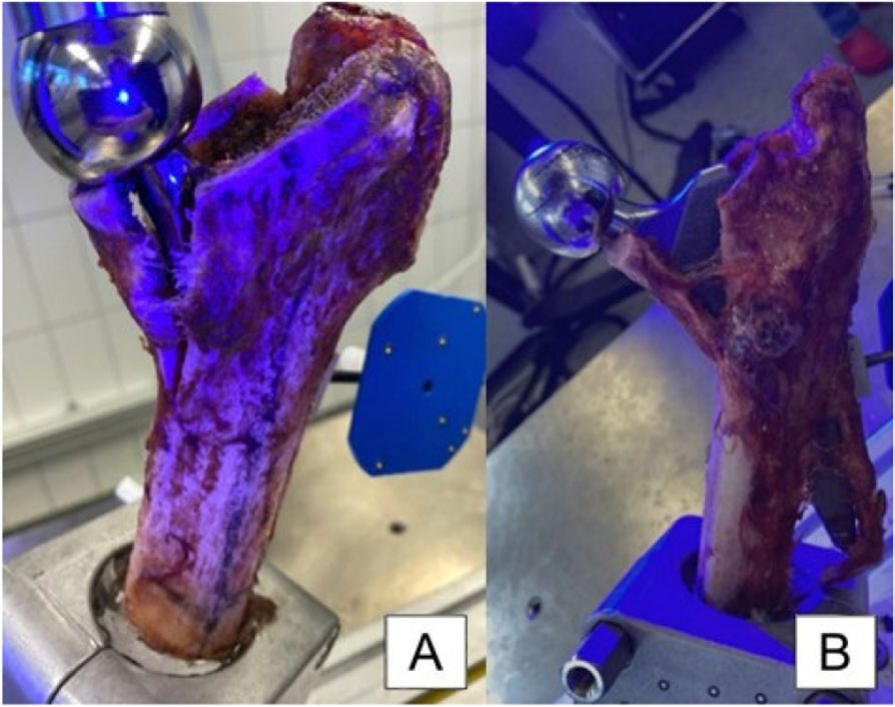
**A** Failure mode of a specimen from Neutral Group post testing, featuring medial wall defect and stem subsidence; (**B**) Failure mode of a specimen from Varus Group post testing, featuring a medial defect accompanied by a lateral wall blow of the stem along with stem subsidence

Implant size in the Neutral Group was significantly bigger than in the Varus Group, *p* = 0.002. Load at 0.15 mm subsidence and cycles to 0.15 mm subsidence in the Neutral Group were lower but not significantly different in comparison to the Varus Group, *p* = 0.214. Load at 1 mm subsidence and cycles to 1 mm subsidence in Neutral Group were significantly lower than those in Varus Group, *p* = 0.022 (Fig. 4). Load at catastrophic failure and cycles to catastrophic failure in Neutral Group were significantly lower versus Varus Group, *p* = 0.018. Failure mode was similar in both groups, featuring a fracture pattern with a medial wall defect, sometimes accompanied by a lateral wall blow of the stem and stem subsidence (Fig. 5).

## Discussion

This work aimed to evaluate the stability of a cementless metadiaphyseal anchoring hip stem implanted in neutral or 8° varus position in a paired study design. To address the question of which position demonstrates better stability, biomechanical testing was carried out applying quasi-static and progressively increasing cyclic loading protocols [18, 19]. The outcomes of the study showed that implant subsidence of 0.15 mm was related to no significant differences between the groups. In terms of subsidence of 1 mm, however, there was a significant advantage in favour of Varus Group. Another interesting aspect was the biomechanical behaviour of the constructs related to the rapid change of the load–displacement curve during dynamic loading, usually reflecting a sudden position change of the prosthesis stem in terms of loosening, followed by a state of regained stability [17]. Here, Varus Group demonstrated a significant advantage compared to Neutral Group despite the use of its smaller implants.

The minimally invasive surgical technique in hip arthroplasty is known for posing the risk of varus malalignment of a cementless stem, and clinical consequences are described differently regarding short- and long-term outcomes. Whilst the risk of intraoperative fractures resulting from a varus malposition of the stem is well described, the long-term outcomes of such a variation in the position do not seem to affect good clinical results [14, 20, 21]. Moreover, there is evidence that varus malalignment of a cementless metadiaphyseal anchoring hip stem not only does not increase the risk of a perioperative fracture but even decreases it compared to the same stem brought in a neutral position [20] The only described disadvantages related to varus malalignment of the stem with regard to the long-term outcomes can be expressed with increased stress shielding, risk of stem undersizing, as well as risk of hip offset [13, 21]. The current work focused on the effect of varus malalignment and consecutive undersizing of the implant dimension, along with the consequences for primary axial stability in a biomechanical setting. The difference in implant size was 1.7 on average, being significantly bigger in the Neutral Group. This difference was found to be higher compared to previous similar evidence investigating a size difference of 1 [14]. Whilst the implant size and position were considered as independent parameters in a study of Konow et al., both were interdependent parameters in the current work. We believe that the selected implant size must depend on its position to better accommodate the clinical reality. In agreement with the work of Konow et al., the results from the current study revealed superior stability of the varus-positioned implants at 0.15 mm and especially at 1 mm stem subsidence—the latter with indicated significance. This constitutes a novel finding, as Konow et al. reported only non-significantly increased stability of varus-positioned stems [14]. Nevertheless, the focus of our work was set on the clinically relevant thresholds of 0.15 mm and 1 mm subsidence. The former was defined as a critical cut-off for press-fit implant micro-instability by Bieger et al., whereas the latter was set as a cut-off for macro-instability for press-fit implants by Kastner et al. [16, 17]. Konow et al. noticed that varus-positioned stems demonstrated higher primary stability and significantly higher micromotions (0.017 mm for varus-positioned stems versus 0.008 mm for valgus and neutrally oriented stems) [14]. In contrast, our results demonstrate significantly higher values for stems in neutral position. Furthermore, the current study did not detect significant differences between the groups for the parameters of interest related to 0.15 mm stem subsidence. In our opinion, this results from the modern design of the stem used in the current study, featuring a larger profile and superior characteristics of the surface coating. Obviously, a varus stem malposition is related to bigger micromotions and decreased axial stiffness compared to a neutral orientation. However, these micromotions led to a similar stability in both groups until subsidence of 0.15 mm occurred. Between 0.15 mm and 1 mm subsidence, the stability in Varus Group significantly increased in comparison to Neutral Group, so that the varus malposition led to a decisive advantage in favour of the former, as the stem achieves more robust cortical engagement during subsequent subsidence. Whilst some authors claim a coherence between a varus-aligned stem and an increased risk of periprosthetic fractures, others report different results [14, 15, 20, 22]. During our study, we did not notice an increased risk of a periprosthetic femoral fracture caused by a varus-aligned stem. Not only did Varus Group show superior biomechanical stability, but a similar mode of failure was observed in both groups, with fracture patterns mostly expressed in terms of a lateral wall blowout of the stem without significant affection of the medial wall. Our outcomes reflect recent evidence, indicating that an intraoperative position change of an initially varus-aligned stem is not worth because of the risk of a potential fracture due to implant removal and realignment [20]. Considerably more can a varus-aligned stem be seen as implementing an inherent useful quality to control hip offset and stability of the joint [22]. The superior resistance to subsidence observed in varus-aligned stems appears to be a multifactorial phenomenon. While the current data cannot definitively distinguish between the individual effects of 8° angulation, implant undersizing, and specific cortical contact points, it is our view that the modification of the fixation pattern and the resulting altered fixation strength are the primary drivers of the observed stability. However, the precise weight of each of these contributing factors needs to be investigated in further studies.

This study has some limitations inherent to all human cadaveric investigations using a limited number of specimens without the possibility to fully simulate complex in vivo situations. Our data are biomechanical and do not serve as a clinical recommendation for intentional varus positioning. Lacking soft tissue simulations weakens the findings and suggestions for clinical application. Precision regarding specimen preparation and implantation revealed variations due to a manually crafted experimental setup. Long-term osseointegration and stress shielding were not evaluated; therefore, our biomechanical results cannot be directly extrapolated to long-term clinical outcomes. The strengths of the present work are expressed via the relatively large sample size featuring anatomically matched, pairwise assigned femora. Original stems with digital planning before implantation were used to ensure optimal size selection for each specimen.

## Conclusions

An 8° varus-aligned cementless metadiaphyseal anchoring hip stem demonstrates superior load-bearing capacity with higher loads and numbers of cycles until reaching defined subsidence thresholds under dynamic loading, as compared to neutral alignment. These results demonstrate the biomechanical tolerance of unintended intraoperative varus malalignment, but do not support or recommend intentional varus stem positioning. Although biomechanical findings indicate sufficient primary stability of varus-aligned stems, surgeons must weigh these against clinical risks.

## Data Availability

The datasets used and/or analysed during the current study are available from the corresponding author on reasonable request.
